# A Minimally Invasive Framework Reveals Region‐Specific Cerebrovascular Remodeling in Aging Using Intravital Functional Ultrasound Imaging and Ultrasound Localization Microscopy (fUS‐ULM)

**DOI:** 10.1002/advs.202510754

**Published:** 2025-09-14

**Authors:** Sharon Negri, Adam Nyul‐Toth, Madison Milan, Eva Troyano‐Rodriguez, Sherwin Tavakol, Jennifer Ihuoma, Zeke Reyff, Rakesh Rudraboina, Rafal Gulej, Raymond Jang, Anna Csiszar, Zoltan Ungvari, Audrey Cleuren, David R Miller, Andriy Yabluchanskiy, Mickael Tanter, Stefano Tarantini

**Affiliations:** ^1^ Vascular Cognitive Impairment and Neurodegeneration Program, Reynolds Oklahoma Center on Aging/Center for Geroscience and Healthy Brain Aging Dept. of Neurosurgery OUHSC Oklahoma City OK 73104; ^2^ Stephenson Cancer Center OUHSC OKC Oklahoma City OK 73104 USA; ^3^ Casady High School Oklahoma City OK 73120 USA; ^4^ Oklahoma Medical Research Foundation Oklahoma City OK 73104 USA; ^5^ Stephenson School of Biomedical Engineering University of Oklahoma Norman OK 73019 USA; ^6^ Institute Physics for Medicine Paris, INSERM U1273, ESPCI PSL Paris PSL Research University Paris 75015 France

**Keywords:** brain, ICONEUS, live animal, live imaging, microvasculature

## Abstract

Aging impairs cerebrovascular structure and function, contributing to cognitive decline and dementia. Here, a novel, high‐resolution, intravital imaging platform is presented that combines functional ultrasound (fUS) and ultrasound localization microscopy (ULM) through a chronically implanted, polymethylpentene (TPX) cranial window, a transparent implant that enables ultrasound imaging through the skull. This approach enables intravital, longitudinal, minimally invasive assessment of cerebrovascular structure and function across cortical and deep brain regions. Leveraging this platform, a new method is developed to estimate resting cerebral blood flow (CBF) by integrating microbubble (MB) velocity data from fUS with microvascular geometry derived from ULM. Notably, a significant age‐related decline in the cortical arteriole‐to‐venule ratio (AVR) is discovered, introducing a novel biomarker of structural cerebrovascular remodeling. It is also validated that fUS can reliably assess neurovascular coupling (NVC) responses in aged mice. This study establishes a powerful, non‐invasive, and repeatable investigative tool for future preclinical studies aimed at evaluating the efficacy of therapeutic interventions targeting vascular contributions to cognitive impairment and neurodegeneration.

## Graphical abstract

A high‐resolution intravital imaging platform combining functional ultrasound and localization microscopy enables longitudinal, minimally invasive assessment of cerebrovascular function in aging studies. The present investigation reveals aging‐related reductions in neurovascular coupling, arteriole‐to‐venule ratio and vascular density, introducing a powerful approach to investigate cerebrovascular remodeling and its contribution to cognitive decline and neurodegeneration.

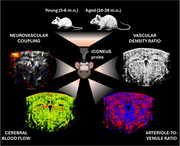

## 1 Introduction

The aging process is accompanied by significant alterations in cerebrovascular structure and function, which have profound implications for cognitive health. These vascular changes are critical contributors to the development of vascular cognitive impairment and dementia (VCID), a prevalent condition among the elderly population.^[^ ^1^, ^2^, ^3^, ^4^, ^5^ ^]^ VCID is linked to impaired blood flow in the brain, often caused by cerebrovascular dysfunctions such as stroke or small vessel disease, and their prevalence is rising as the elderly population grows.^[^ ^6^, ^7^ ^]^ VCID represents a spectrum of cognitive disorders ranging from mild deficits to full‐blown dementia, contributing substantially to the global burden of dementia, which in 2020 affected over 55 million people worldwide.^[^ ^8^ ^]^ This number is expected to nearly double every 20 years, reaching 78 million by 2030 and 139 million by 2050 (https://www.who.int/news‐room/fact‐sheets/detail/dementia). The importance of cerebrovascular health in aging cannot be overstated, as compromised vascular function accelerates cognitive decline.^[^ ^9^, ^10^ ^]^ The understanding of vascular remodeling, including rarefaction, and neurovascular coupling (NVC) in the maintenance of brain's ability to function, adapt, and repair, has become crucial in studying the prevention of vascular‐related cognitive decline.^[^ ^11^, ^12^ ^]^ Understanding the mechanisms underlying age‐related cerebrovascular remodeling is essential for developing strategies to mitigate cognitive decline associated with aging.^[^ ^13^ ^]^ One prominent feature of cerebrovascular aging is the reduction in vascular density, known as vascular rarefaction. Studies utilizing high‐resolution imaging techniques have demonstrated a decrease in vascular length and branching density in the aging mouse brain, particularly affecting deep cortical layers and basal forebrain regions.^[^ ^14^ ^]^ This decline in microvascular networks can lead to diminished cerebral blood flow (CBF), resulting in inadequate oxygen and nutrient delivery to neural tissues, thereby contributing to cognitive impairments.^[^ ^15^ ^]^ In addition to structural changes,^[^ ^16^ ^]^ aging is associated with functional impairments in NVC, the mechanism by which neuronal activity dynamically regulates local blood flow. Research indicates that NVC responses are significantly attenuated in aged mice, suggesting a diminished capacity of the neurovascular unit to meet the metabolic demands of active neurons.^[^ ^17^, ^18^, ^19^ ^]^ Such impairments may exacerbate neuronal stress and contribute to the pathogenesis of neurodegenerative diseases. The advent of functional ultrasound (fUS) imaging has provided a powerful tool for non‐invasively assessing cerebrovascular function with high spatiotemporal resolution. fUS imaging enables the visualization of cerebral blood volume (CBV) changes as an indirect measure of neuronal activity, facilitating the study of NVC dynamics in both anesthetized and awake animal models.^[^ ^20^, ^21^, ^22^ ^]^ This technique has been instrumental in advancing our understanding of the complex interplay between vascular and neural components in the aging brain.^[^ ^23^ ^]^ The advent of fUS imaging marks a major advancement in neuroscience, enabling in vivo investigation of the cerebrovasculature within the cortex and providing valuable insights into the brain's blood supply and its relationship with neural function.^[^ ^24^ ^]^ Two‐photon microscopy imaging allows researchers to observe and analyze the cortical vasculature in real time, enabling a better understanding of blood flow dynamics and how they impact brain health and disease.^[^ ^3^, ^25^, ^26^ ^]^ However, studying deeper cerebral structures, like the hippocampus, presents unique challenges. The complexity of these structures, coupled with their location beneath multiple layers of cortical tissue, makes it difficult to access using traditional imaging methods,^[^ ^27^, ^28^, ^29^ ^]^ characterized by a limited penetration depth of light in optical imaging and the difficulty of achieving high‐resolution imaging at greater depths. Moreover, it has long been known that the vascular remodeling occurring in aging is heterogeneous across the whole brain, meaning that a different technique is required to fully comprehend this variability.^[^ ^30^, ^31^ ^]^ The growing adoption of fUS imaging has recently emerged as a powerful tool to overcome the technical challenges associated with studying deep cerebral structures. Unlike traditional optical imaging techniques, fUS utilizes high‐frequency sound waves to visualize the entire brain vasculature in vivo, regardless of depth. This breakthrough allows for the dynamic observation of blood flow in both cortical and deeper regions of the brain, including the hippocampus. By providing high spatial resolution and the ability to monitor blood flow in real time, fUS imaging addresses the limitations of optical methods, enabling researchers to gain a comprehensive understanding of the cerebrovasculature across all regions of the brain.

A key development enhancing the capabilities of fUS is Ultrasound Localization Microscopy (ULM), a super‐resolution technique that leverages intravenously injected microbubbles (MBs) as contrast agents.^[^ ^32^, ^33^ ^]^ By tracking the trajectories of individual MBs over time, ULM enables precise mapping of microvascular structures far beyond the diffraction limit of conventional ultrasound. This approach allows for unprecedented visualization and quantification of the cerebral microvasculature, including capillary‐level details, which are critical for assessing age‐related vascular remodeling and NVC. ULM thus offers a powerful, noninvasive method to detect subtle cerebrovascular changes that may underline cognitive decline and neurodegenerative diseases.^[^ ^28^, ^34^ ^]^ To facilitate repeated, high‐resolution imaging of the mouse brain with minimal invasiveness, we combined ULM with the implantation of a transparent plastic cranial window made of polymethylpentene (TPX). This chronic imaging window was specifically developed to overcome the limitations of traditional cranial windows, such as bone regrowth, signal attenuation, and poor acoustic coupling. TPX is an ultrathin, biocompatible, and acoustically transparent polymer that permits stable transmission of ultrasound waves while maintaining optical clarity. Its mechanical properties allow for long‐term implantation without compromising animal health or imaging fidelity. The TPX window provides a stable and reusable platform for longitudinal in vivo imaging of cerebrovascular structure and function using fUS and ULM techniques, enabling detailed assessments of vascular remodeling across time in both young and aged animals.^[^ ^28^, ^34^ ^]^

**Figure 1:**
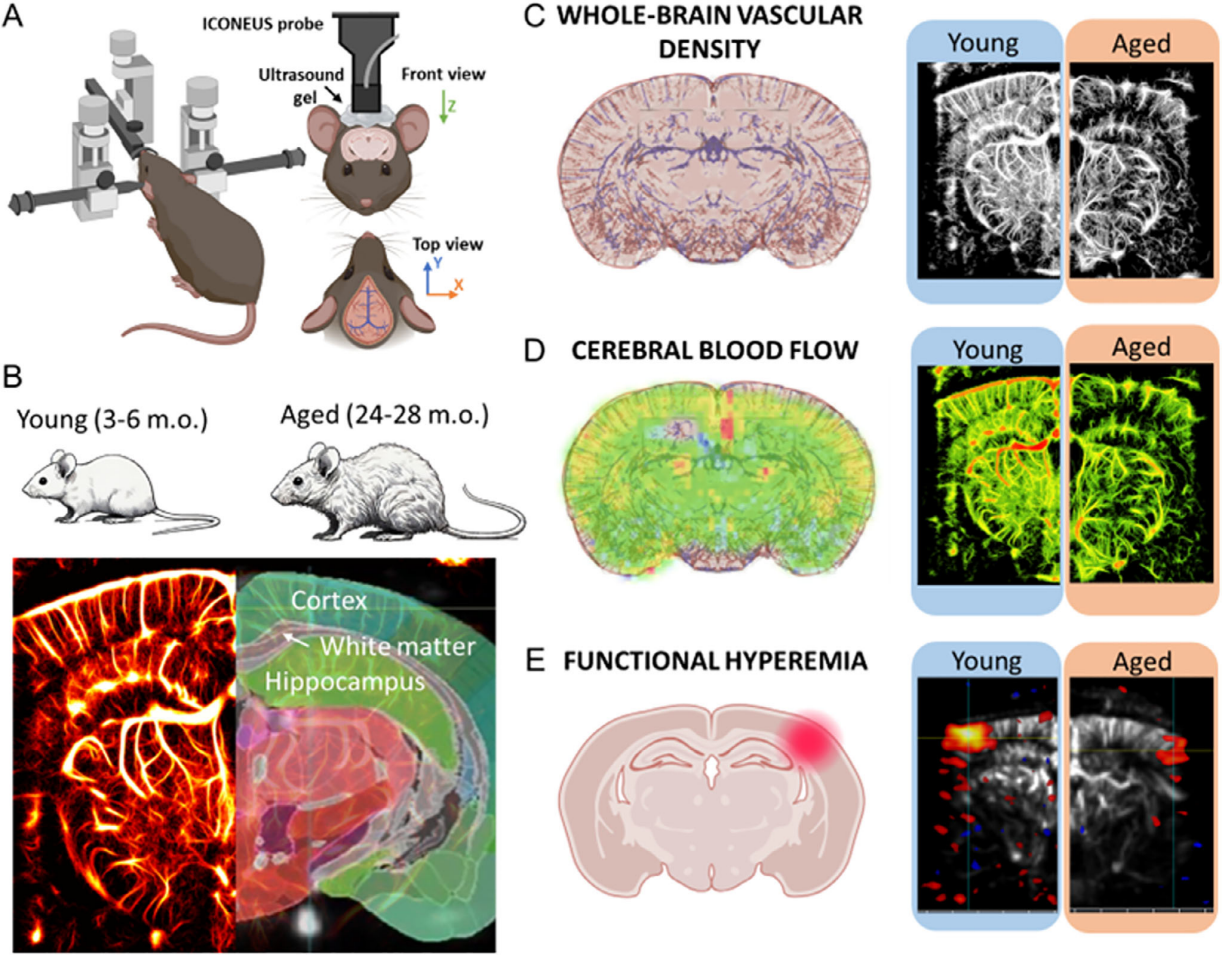
Summary of neurovascular imaging with ICONEUS fUS imaging. A) Schematic representation of the experimental setup for the fUS imaging with ICONEUS. B. Coronal planes of young (3–6 months old) and aged (24–28 months old) mice were imaged during whisker stimulation and in resting conditions after contrast agent (MBs) injection. Example of the resulting ULM maps with the superimposition of the mouse brain atlas. In this study, we were interested in cortex, hippocampus, and subcortical white matter. C) ULM maps were used to evaluate the vascular coverage in young and aged mouse brains. D) Evaluation of resting CBF, schematic representation (left), and ICONEUS‐produced images in young and aged mice. The resting CBF was calculated by considering the vessels’ diameter and the speed in each pixel of the image. E) Measurement of functional hyperemia following the contralateral whiskers stimulation.

In this study, we uniquely combined ULM with a chronically implanted TPX cranial window to assess cerebrovascular network structure and function in young and aged mice. This integrated approach enables high‐resolution, longitudinal imaging of previously inaccessible brain regions, providing new insights into age‐related changes in cerebral microcirculation and their implications for brain function and pathology. Given the critical role of cerebrovascular health in cognitive function, it is imperative to elucidate the mechanisms by which aging influences vascular structure and function. This study aims to investigate the impact of aging on cerebrovascular parameters, including vascular density ratio, vessel diameter, CBF dynamics, and NVC responses, utilizing fUS imaging in an aged mouse. By comprehensively characterizing these age‐related changes, we seek to contribute to the development of therapeutic strategies aimed at preserving cognitive function in the aging population (**Figure** **1**).

## 2 Results

### 2.1 Cortical, Hippocampal, and White Matter Vascular Coverage Declines with Aging

**Figure 2:**
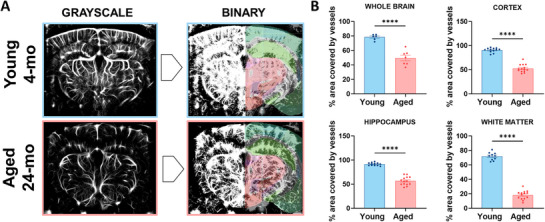
Age‐Related decrease in vascular coverage can be quantified in vivo with fUS imaging. A) ICONEUS‐produced greyscale images were converted into binary images through a costumed MATLAB script. Vascular coverage was evaluated in different portions of the young and aged mice's brain (i.e., whole brain, cortex, hippocampus, and white matter). Mouse brain atlas is superimposed for a better understanding. B) Statistical quantification of vascular coverage evaluated by percentage of colored pixels. Aged mice showed a significantly reduced vascular density ratio in all four regions investigated. Data are expressed as mean ± SEM. **** indicate *p* < 0.0001. Statistical significance was evaluated by the Unpaired T‐test.

To assess vascular remodeling in aged brains compared to young brains, mice were implanted with a TPX window as described previously^[^ ^34^ ^]^ and allowed a 14‐day recovery period. Following recovery, mice were anesthetized, shaved, and secured in a stereotaxic frame beneath the fUS imaging probe. The probe was positioned directly above the brain and immersed in ultrasound gel to enable image acquisition. Greyscale ULM images were acquired by injecting the animals with 100 µL of MBs solution, which served as a contrast agent to facilitate vessel detection. The movement of these MBs was recorded over a 10‐min period. Greyscale images were processed using proprietary ICONEUS software and converted to binary vessel maps using custom MATLAB code. We quantified the percentage of positive (vessel‐containing) pixels in the whole brain and in predefined regions of interest (ROIs), including the cortex, hippocampus, and white matter. These regions were selected due to their essential roles in higher‐order cognitive functions, including memory and learning. Notably, the white matter plays a key role in interregional connectivity, and age‐related declines in its vascularization have been linked to increased stroke risk and cognitive impairment.^[^ ^35^, ^36^ ^]^ To ensure accurate regional analysis, the Allen Brain Atlas was co‐registered with the binary vascular maps (**Figure** **2**A) based on automatic detection of the animal vascular print.^[^ ^37^ ^]^ based on an automatic detection of the animal vascular print and comparison with the vascular print of reference animals. The accuracy of this automatic Brain Positioning System (BPS) was studied in a former work,^[^ ^38^ ^]^ and positioning errors of 44 and 96 µm, respectively, for intra‐animal and inter‐animal vascular registration were found. The BPS approach outperforms the manual vascular landmark recognition performed by expert neuroscientists (inter‐annotator errors of 215 and 259 µm).^[^ ^38^ ^]^ Quantitative analysis revealed a significant reduction in vascular density across the whole brain, as well as in the cortex, hippocampus, and white matter of aged mice compared to young controls (Figure 2B).

### 2.2 Aging Leads to Narrowing of Microvessels in Key Brain Regions

**Figure 3:**
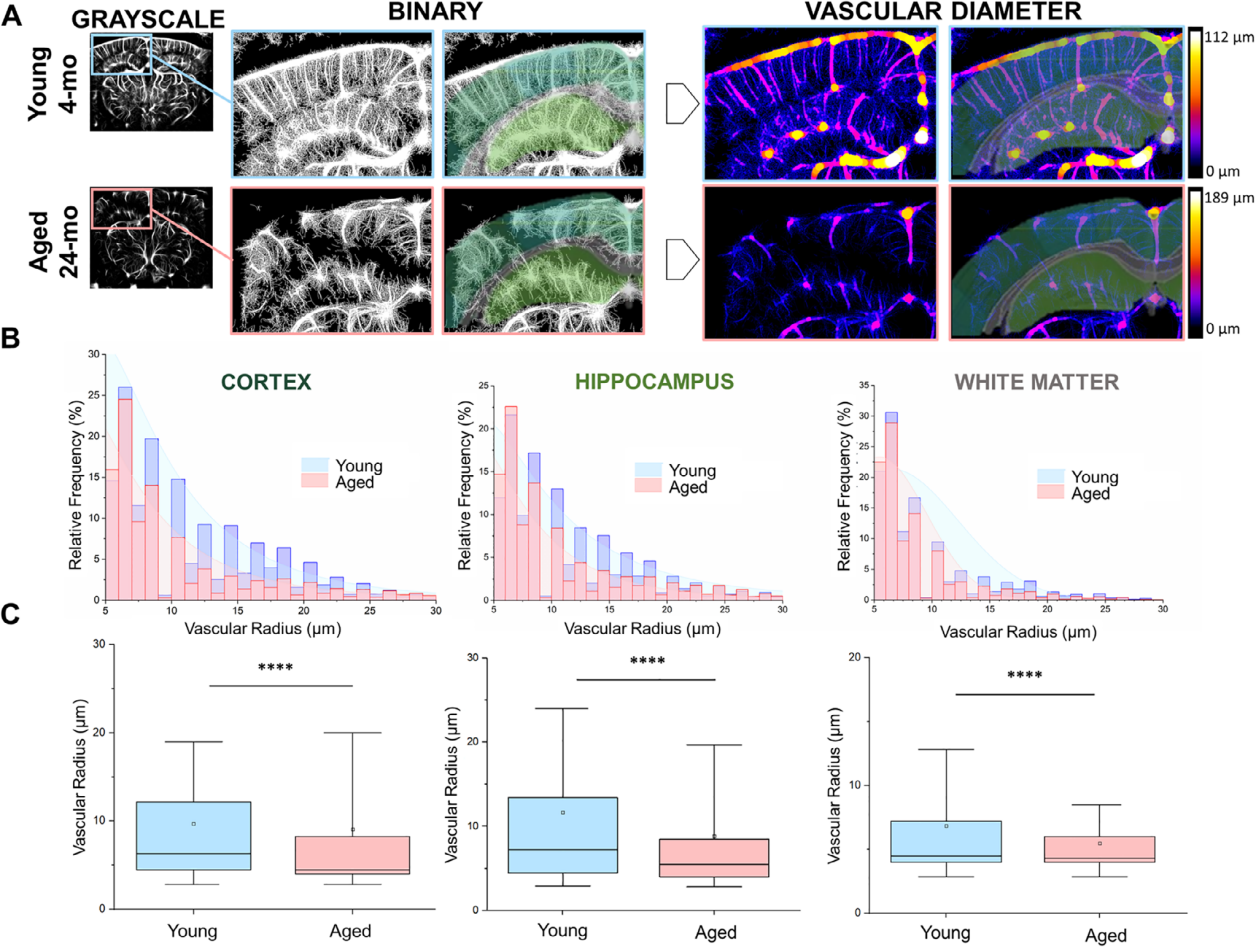
Brain vascular diameter is reduced by aging in the somatosensory cortex, hippocampus, and subcortical white matter. A) The vascular diameter color map is generated from the binary image using the Local Thickness function in FIJI. The color scale bars in panel A are provided for visual reference only; all quantitative analyses were performed on normalized pixel values, ensuring consistent comparison across groups. B) The relative frequency distribution of vessel diameters in the cortex, hippocampus, and subcortical white matter. C) Boxplot representation of data distribution across the mentioned brain regions. The box represents the 25th‐75th percentile, the empty square represents the average, and the whiskers indicate the 10th‐90th percentile range. Statistical significance was evaluated using Welch's t‐test, which does not assume equal variance between groups. **** denotes *p* < 0.0001.

To investigate the impact of aging on microvascular architecture, we analyzed the distribution of vascular diameters in young and aged mouse brains. Binary images of the vasculature were processed using the Local Thickness function in FIJI, generating color‐coded representations where purple indicates smaller vessels and yellow denotes larger vessels (**Figure** **3**A). This visualization revealed a higher prevalence of larger vessels in young brains, especially within the cortex and hippocampus. In contrast, aged brains exhibited a shift toward a greater proportion of smaller vessels in these regions (Figure 3B). Quantitative analysis demonstrated a significant reduction in average vascular diameter with aging across the cortex, hippocampus, and white matter (Figure 3C). These findings align with previous research indicating that aging is associated with microvascular rarefaction and structural remodeling, leading to diminished vessel calibers and increased vascular tortuosity.^[^ ^39^, ^40^, ^41^, ^42^ ^]^

### 2.3 Aging Alters Regional Estimated CBF Velocity

**Figure 4:**
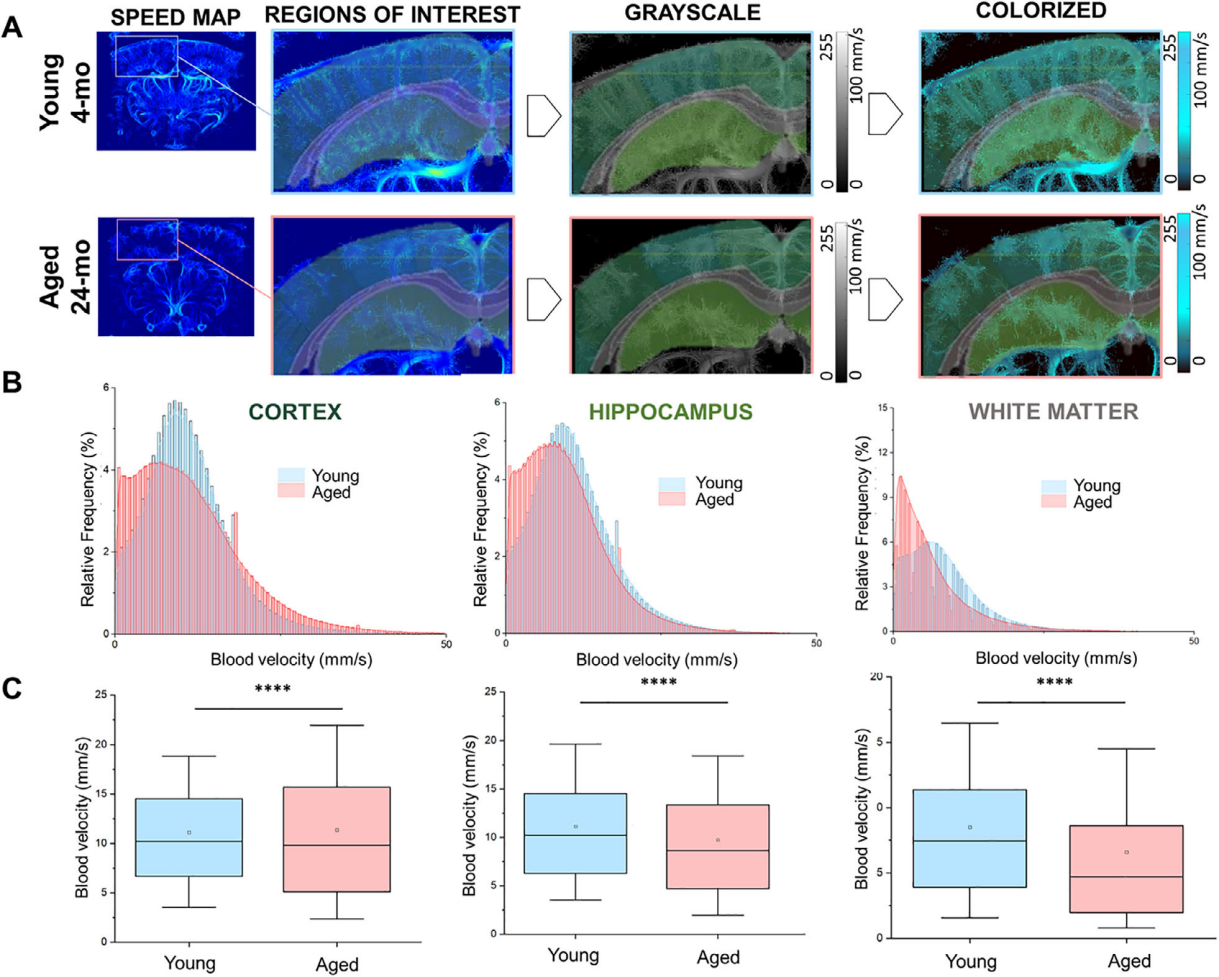
Age‐dependent change in CBF speed in cortex, hippocampus, and white matter. A) Speed map images are produced by ICONEUS software based on the speed of the MBs flow between 0 and 100 mms^−1^. Images were converted into greyscale (0 to 255) images for the quantification through a MATLAB script, and the obtained values were then divided by 2.55. A different LUT was then utilized to better characterize the speed variation toward the brain. B) Relative frequency distribution of the speed n cortex, hippocampus, and subcortical white matter in young and aged animals. C) Boxplot representation of the data distribution in the brain regions mentioned. The box represents the 25th‐75th percentile, the empty square represents the average, and the whiskers indicate the 10th‐90th percentile range. Statistical significance was evaluated using Welch's t‐test, which does not assume equal variance between groups. **** denotes *p* < 0.0001.

**Figure 5:**
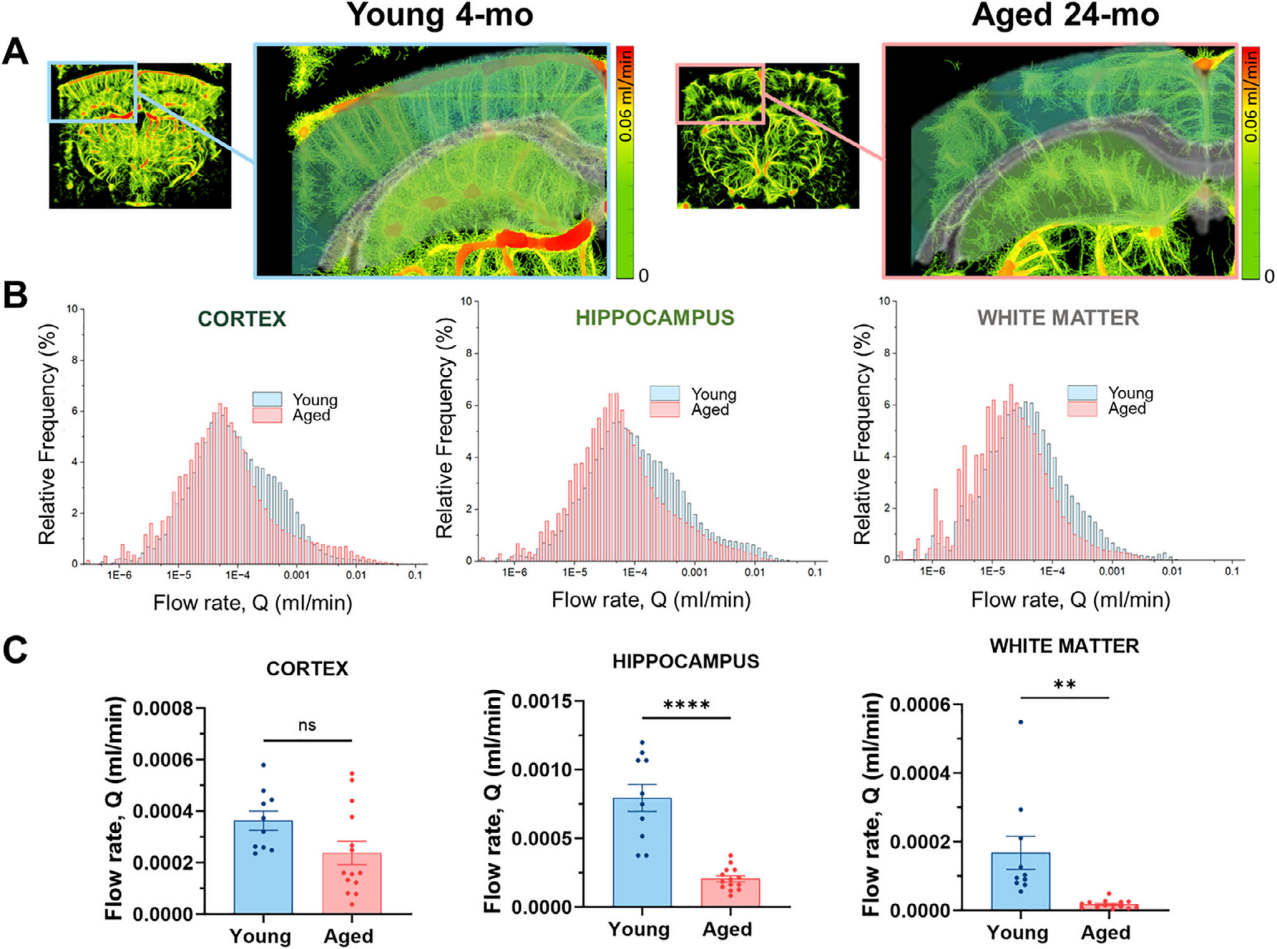
Resting CBF rate (Q) is significantly reduced in hippocampus and white Matter during aging. A) Representative color coded images of the flow rate in aged and young brains. Images were obtained by combining the local thickness images and speed map from the same brain through a MATLAB script. B) Relative distribution of resting blood flow rate in young and aged cortex, hippocampus, and white matter. C) Quantification of resting CBF rate in young and old mice in the whole brain, cortex, hippocampus, and white matter. Data are shown as mean ± SEM. Statistical significance was evaluated by Unpaired T‐test. **** indicate *p* < 0.0001; ** indicate *p* < 0.005.

Speed maps (blue images) exported from ICONEUS software were used to calculate blood flow velocity in the cortex, hippocampus, and white matter. The blue images were converted to greyscale images, and a blue LUT was applied for better visualization (**Figure** **4**A). Blood flow velocities between 0 and 100 mms^−1^ were considered, and the relative distribution of blood flow velocity in the cortex, hippocampus, and white matter was plotted (Figure 4B). Interestingly, a shift towards higher velocity was observed in the aged brain cortex compared to the young cortex. In contrast, the pattern was reversed in deeper regions such as the subcortical white matter and hippocampus (Figure 4B). Statistical analysis confirmed an increase in velocity with aging in the cortex, while a significant reduction was found in the hippocampus and white matter (Figure 4C). Data are presented as box plots (25th‐95th percentiles; whiskers 10th‐90th percentiles, with the mean indicated by an empty white box). Similarly, the resting CBF rate, calculated both mathematically and visually as described in the Materials and Methods, was significantly decreased by aging in the hippocampus and white matter, but not in the cortex (Figure 5C). This finding of a preserved cortical CBF aligns with the significant decrease in local vessel diameter in the cortex and the opposite increase in blood velocity in this region. Such a non‐significant change in resting cortical CBF rate could be potentially interpreted as a preserved cerebral autoregulation in this region with aging. To visualize these changes, CBF maps were generated by merging local thickness images with greyscale speed maps using MATLAB, following the Poiseuille formula (**Figure** **5**A). The relative distribution of flow rates was computed for each pixel based on the combined data from local thickness and speed map images (Figure 5B).

### 2.4 Aging Significantly Decreases the Penetrating Arteriole‐to‐Venule Ratio (AVR) in Brain Cortex

**Figure 6:**
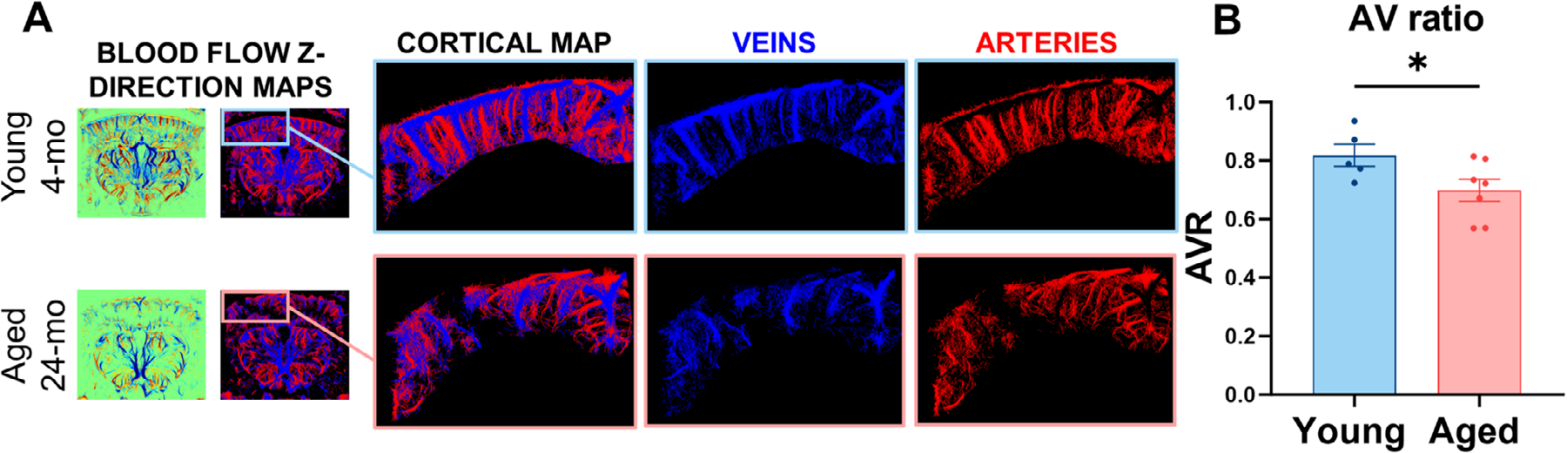
AVR is significantly reduced by aging. A) Representative Y‐directional blood flow maps in aged and young mice were exported by ICONEUS software. The images were then processed with FIJI to isolate the arterioles (red) from the venules (blue). B) Quantification of AVR was performed with a MATLAB script. The bar graph showed a significant decrease in the AVR in aged mice compared to young ones. Data are shown as mean ± SEM. Statistical significance was evaluated by Unpaired T‐test. * indicates *p* < 0.05.

To investigate the impact of aging on the balance between arterial and venous vasculature in the mouse brain cortex, we analyzed the flow direction of the MBs in the generated ULM maps. These maps allowed us to distinguish between penetrating arterioles and venules by identifying MBs moving downward (arteriolar flow, shown in red) and upward (venular flow, shown in blue) using FIJI processing techniques (**Figure** **6**A). The mouse cortical AVR was quantified utilizing a custom MATLAB script, which revealed a significant reduction in this ratio in aged mice compared to their younger counterparts (Figure 6B). Although the retinal AVR is a well‐established clinical marker of microvascular health, an equivalent measure within the cerebral cortex has not been systematically explored. With fUS, we quantified cortical AVR in mice and found that it is significantly altered with aging, revealing a novel potential structural biomarker of cerebrovascular remodeling. This observed decrease in the mouse cortical AVR suggests a disproportionate decline in arterial vessels relative to venous vessels within the cortical regions of aged mice.

### 2.5 Stimulus‐Evoked Functional Hyperemia is Significantly Reduced with Aging

**Figure 7:**
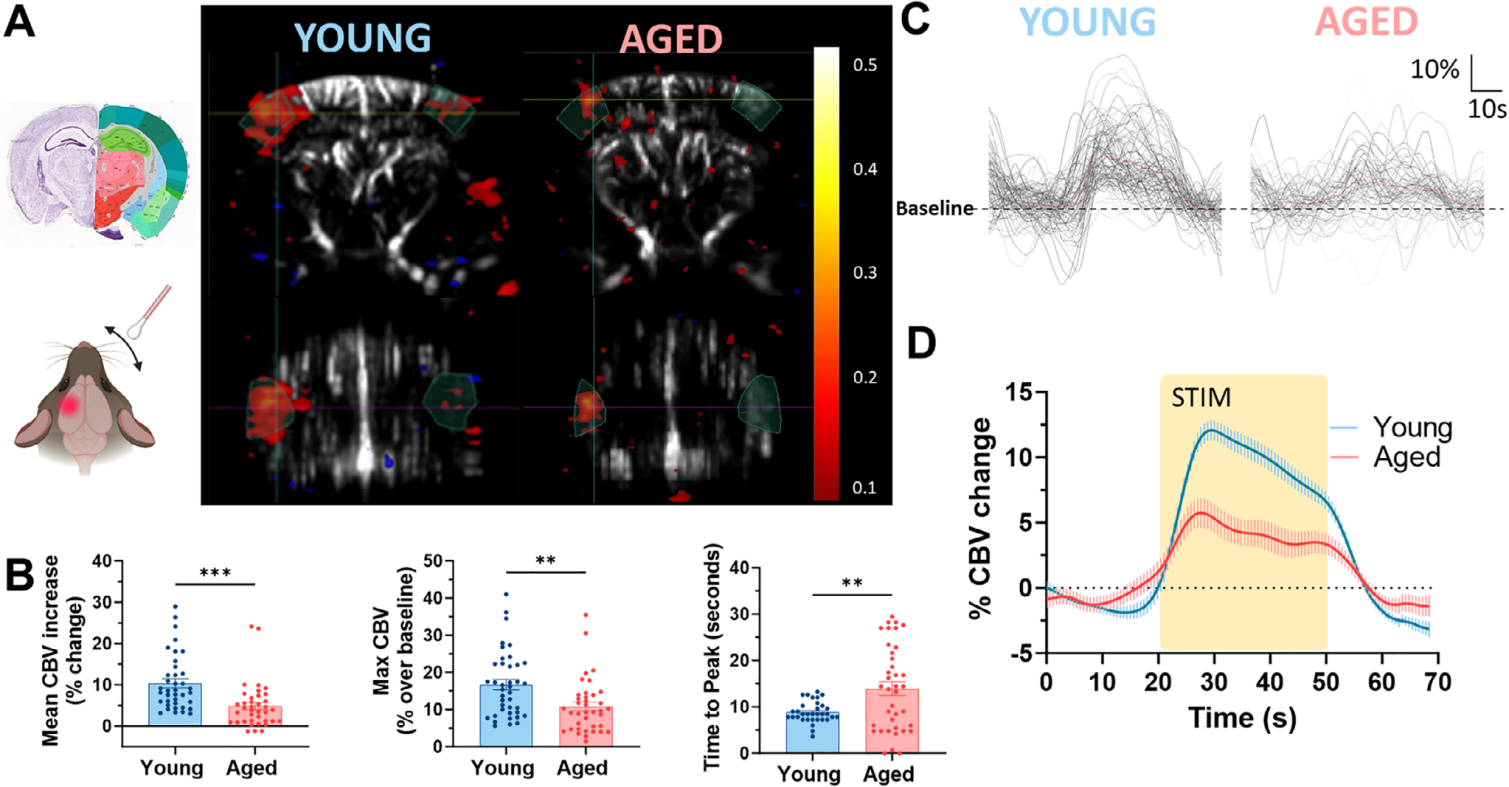
NVC is significantly reduced in aged animals compared to young ones. A) NVC is defined as an increase in CBF in response to the activation of a specific brain area. NVC responses in young and aged mice are represented in coronal (upper box) and axial (lower box) sections in response to contralateral whisker stimulation. B) Bar charts of average response and peak values clearly showed a reduction of the NVC responses in aged mice. On the contrary the time to peak was significantly longer in aged mice compared to young ones. Data are shown as mean ± SEM. Statistical significance was evaluated by an unpaired t‐test on 38 recorded responses in aged mice and 33 in young mice. *** indicate p < 0.001; ** indicate p < 0.005. C) Graphical representation of all the recorded responses to 30 s of stimulation in young (left plot) and aged (right plot) animals. D) Average curve of responses in young (blue curve) and aged (red curve) animals. All the different representations clearly show how the NVC responses are significantly reduced in aged mice compared to young ones. The yellow rectangle indicates the whisker stimulation time window.

NVC is a critical mechanism by which neuronal activity regulates CBF to meet metabolic demands. Previous studies from our group demonstrated age‐related impairments in NVC using laser speckle contrast imaging (LSCI).^[^ ^43^ ^]^ In this study, we employed fUS imaging to further investigate and validate these findings, offering enhanced spatial resolution and depth penetration. We assessed functional hyperemia by measuring baseline CBF for 30 s, followed by 30 s of whisker stimulation, and then a 30‐s post‐stimulation recording. This cycle was repeated seven times to ensure reproducibility. The resulting CBF changes in the contralateral somatosensory cortex were analyzed. Representative fUS images illustrate the magnitude of the hemodynamic response, with the dimension and intensity of the red regions indicating the extent of CBF increase in both coronal and axial planes (**Figure** **7**A). Statistical analysis revealed a significant reduction in both the average CBF response and peak values in aged mice compared to young mice (Figure 7B). Additionally, the time required to reach peak CBF was notably prolonged in aged animals, suggesting a delay in the vascular response to neuronal activation. Individual CBF traces (Figure 7C) and averaged response curves (Figure 7D) further highlight the diminished and delayed NVC responses in aged mice. These findings are consistent with previous reports indicating that aging is associated with a decline in NVC efficiency and confirm the ability of fUS imaging to quantify the longitudinal decrease of NVC response with normal or pathological aging.^[^ ^44^ ^]^

## 3 Discussion

Our study utilized fUS imaging to investigate age‐related cerebrovascular changes in mice, revealing significant reductions in vascular density ratio, CBF, and NVC in aged animals. These findings contribute to the growing body of evidence indicating that aging adversely affects cerebrovascular integrity and function,^[^ ^9^, ^45^, ^46^, ^47^, ^48^ ^]^ while also adding to the validation of fUS imaging as a reliable, non‐invasive method for longitudinal assessment of cerebrovascular function.^[^ ^34^, ^49^ ^]^ The observed decrease in vascular density ratio aligns with previous research demonstrating microvascular rarefaction in aged mice.^[^ ^2^, ^50^, ^51^ ^]^ The relationship between the vascular density ratio measured by ULM and actual vascular density has been validated through ex vivo histological analysis, as recently demonstrated in our study,^[^ ^52^ ^]^ supporting the accuracy of the in vivo measurements presented here. Such vascular remodeling can lead to diminished perfusion and oxygenation of neural tissues, potentially contributing to cognitive decline.^[^ ^3^, ^40^, ^53^, ^54^ ^]^ Our CBF measurements were based on a simplified Poiseuille‐derived model that combines vessel radius and blood velocity to calculate volumetric flow rate (mLmin^−1^), enabling regional quantification of perfusion changes with aging. This physiologically informed approach adds a quantitative layer to our fUS imaging and strengthens the interpretation of observed reductions in hippocampal and white matter perfusion. Furthermore, the reduction in CBF observed in our study is consistent with findings from other studies reporting decreased CBF in pre‐clinical and clinical studies.^[^ ^55^, ^56^, ^57^, ^58^, ^59^ ^]^ Impaired NVC, as evidenced by diminished functional hyperemia in response to whisker stimulation, further underscores the compromised cerebrovascular function associated with aging. Our measurements indicate a decline in CBF velocity and flow rate in aged mice, particularly in the hippocampus and white matter regions. Interestingly, while the cortex exhibited increased blood flow velocity, this did not translate to an overall increase in CBF rate, suggesting compensatory mechanisms that may be insufficient in maintaining adequate perfusion. Such alterations can impair CBF and contribute to the pathogenesis of age‐related cognitive decline and neurodegenerative diseases.^[^ ^60^ ^]^ The observed decrease in vascular diameter may be attributed to several factors, including endothelial dysfunction, reduced angiogenic signaling, and accumulation of extracellular matrix components that alter vascular compliance. Notably, studies have reported increased deposition of hyaluronan in the microvasculature of aged mouse cerebral cortex, which could contribute to vessel narrowing and compromised blood flow.^[^ ^61^ ^]^ Furthermore, the hippocampus and white matter are particularly vulnerable to microvascular changes due to their high metabolic demands and complex vascular architecture.^[^ ^62^, ^63^, ^64^, ^65^ ^]^ Age‐related reductions in vessel diameter within these regions can exacerbate hypoperfusion, leading to neuronal dysfunction and white matter lesions commonly observed in aging and dementia.^[^ ^66^, ^67^, ^68^, ^69^ ^]^ The narrowing of vessels in the cortex, hippocampus, and white matter highlights the need for further research into therapeutic strategies aimed at preserving microvascular integrity to support cognitive health in the aging population. These findings are consistent with reports of age‐related changes in CBF dynamics^[^ ^70^, ^71^ ^]^ and highlight the complexity of cerebral hemodynamics during aging, suggesting that aging differentially affects CBF dynamics across various brain regions. The increased blood flow velocity in the aged cortex may represent a compensatory mechanism to maintain adequate perfusion despite structural vascular changes. As aging leads to microvascular rarefaction, residual vessels may accommodate higher flow to maintain tissue perfusion, especially in metabolically active cortical regions. Several studies have shown that aging impairs myogenic autoregulation in cerebral resistance vessels and attenuates vasodilatory capacity, leading to elevated flow velocity and shear stress in surviving vessels.^[^ ^72^ ^]^ In addition, impaired pericyte function^[^ ^73^ ^]^ and increased tortuosity may further exacerbate uneven flow distribution. Recently, altered blood velocity and vessel diameter measurement by ULM in a mice model of small vessel disease (Hereditary Hemmoragic Telangetesia) were found to correlate with pericyte contractility dysfunction.^[^ ^38^ ^]^ Pericyte dysfunction induced a decrease in blood flow velocity and an increase in vessel diameter. MB flow measured by ULM in HHT mice also suggested a compensatory vascular adaptation, as CBF measured by MB flow was found to be preserved in the pericyte‐ dysfunctional group compared to the control group. Here, our results (an increased blood speed in addition to a decrease in vessel diameter) also favor such compensatory vascular adaptation for preserving CBF during aging. These mechanisms may also explain why the most pronounced velocity reduction with aging was observed in the hippocampus and white matter, which are more susceptible to microvascular dysregulation and hypoperfusion. In contrast, the reduced velocities and flow rates in the hippocampus and white matter indicate a decline in perfusion, which could contribute to the vulnerability of these regions to age‐related cognitive decline and neurodegenerative diseases. Notably, previous studies have reported similar reductions in CBF and vascular density ratio in the hippocampus and white matter of aged mice, supporting our observations.^[^ ^14^ ^]^ Our results also demonstrate a significant decline in NVC responses in aged mice, evidenced by reduced magnitude and delayed peak times of functional hyperemia following whisker stimulation. This impairment suggests that the aging neurovascular unit's ability to regulate blood flow in response to neuronal activity is compromised, which could exacerbate neuronal stress and contribute to cognitive decline. For instance, studies have shown that aged mice exhibit reduced CBF responses to sensory stimulation, implicating endothelial dysfunction and diminished nitric oxide bioavailability as contributing factors.^[^ ^57^, ^58^ ^]^ Furthermore, age‐related structural changes in the cerebrovascular network, such as vessel rarefaction and increased tortuosity, may exacerbate impairments in vascular reactivity^[^ ^74^, ^75^ ^]^. The application of fUS imaging in this context not only corroborates prior LSCI findings but also underscores the utility of fUS as a reliable and non‐invasive modality for assessing age‐related changes in neurovascular function. The enhanced spatial resolution and depth penetration of fUS allow for comprehensive evaluation of both superficial and deep brain regions, providing valuable insights into the cerebrovascular alterations associated with aging. The combination of ULM and fUS provides complementary information. Whereas ULM modality was used to assess resting‐state structural and hemodynamic features, including vascular density, vessel diameter, flow velocity, and resting CBF with high spatial resolution across cortical, hippocampal, and white matter regions, fUS imaging was used to assess NVC by capturing stimulus‐evoked changes in CBV in the somatosensory cortex, offering high temporal resolution (sub‐second scale) but with lower spatial resolution than ULM. Interestingly, functional ULM (fULM) was recently found to be also complementary to fUS and ULM imaging in mice models of small‐vessel disease.^[^ ^38^ ^]^ Indeed, fULM was shown to be able to localize the pericytes dysfunction at the arteriolar‐capillary transition (ACT) zone in Endoglin gene KO mice, in other words, the precise vascular compartment affected by this model of small vessel disease. We could add fULM imaging of functional hyperemia to further complete this “vascular‐omic” characterization of cerebrovascular alterations in future aging studies.

In the murine cortex, arteriolar vessels descend vertically into the brain and are primarily responsible for delivering oxygenated blood and essential nutrients to the brain parenchyma, while venous vessels facilitate the removal of deoxygenated blood and metabolic waste products. Inspired by the retinal AVR as a well‐established clinical marker of microvascular health, we proposed and calculated a novel measure within the cerebral cortex that has not been systematically explored yet. Using high‐resolution imaging, we quantified cortical AVR in mice and found that it is significantly altered with aging, suggesting its potential role as a novel structural biomarker of cerebrovascular remodeling in mouse models. A reduction in the cortex AVR could imply a compromised arterial supply, potentially leading to diminished oxygen and nutrient delivery. This imbalance can adversely affect CBF regulation, contributing to cerebrovascular dysfunction and an increased risk of cognitive decline associated with aging.​ These findings align with previous research indicating that aging is associated with structural and functional alterations in the cerebrovascular system, including reduced vascular density and increased vessel tortuosity. Such changes can impair CBF and are implicated in the pathogenesis of age‐related neurological disorders, such as VCID. The significant decrease in the AVR could substantiate the detrimental effects of aging on cerebrovascular integrity and highlights the importance of maintaining vascular health to support cognitive function in the aging population. Future research should validate this structural biomarker and should focus on elucidating the potential mechanisms underlying the selective vulnerability of arterial vessels to aging and exploring potential therapeutic strategies aimed at preserving or restoring the arterial network within the brain cortex. Additionally, longitudinal studies assessing the progression of AVR changes over time could provide valuable insights into the temporal dynamics of cerebrovascular aging and its relationship with cognitive decline.

In summary, our results demonstrate a significant decline in NVC responses with aging, characterized by reduced magnitude and delayed peak times of functional hyperemia. These impairments in CBF regulation may contribute to the increased susceptibility to cognitive decline and neurodegenerative diseases observed in the elderly population. It could also help to better understand and quantify the impact of pathological conditions or behaviors on NVC aging. Recent fUS studies showed the dramatic impact of obesity on NVC at adolescent age compared to normal aging.^[^ ^44^ ^]^ Future research should focus on elucidating the underlying mechanisms of NVC deterioration with age and exploring potential therapeutic strategies to preserve cerebrovascular health and cognitive function in aging individuals. Collectively, our data underscore the importance of longitudinal‐ region‐specific assessments of cerebrovascular function in aging research. The differential alterations in cerebrovascular structure, CBF, and NVC across brain regions highlight the complex interplay between vascular structure and function in the aging brain. These insights are crucial for understanding the pathophysiology of age‐related cognitive decline and for developing targeted interventions aimed at preserving cerebrovascular health in the elderly population. Additionally, our findings corroborate previous research indicating that aging affects the normal structure and function of the neurovascular unit. Our results are in line with studies utilizing fUS to assess cerebrovascular changes in various contexts. For instance, research has shown that fUS imaging can reveal microvascular rarefaction and impaired NVC in models of chemotherapy‐induced cognitive impairment. Additionally, advancements in fUS technology have enabled the visualization of deep vascular structures and perfusion in aging mouse brains, supporting the validity of our methodological approach. Our findings demonstrate significant age‐related impairments in cerebrovascular structure and function, emphasizing the importance of vascular health in aging and its potential role in cognitive decline. The application of fUS imaging offers a powerful tool for non‐invasively assessing these changes, providing valuable insights that could inform interventions aimed at preserving cerebrovascular function in aging populations.^[^ ^76^, ^77^, ^78^ ^]^ Future research should aim to address these limitations by employing longitudinal designs and incorporating awake imaging protocols to better understand the progression of cerebrovascular changes during aging. Combining fUS with other imaging modalities, such as functional MRI or positron emission tomography, could provide a more comprehensive assessment of neurovascular function.^[^ ^79^ ^]^ In future studies, full depth‐resolving capabilities of fUS/ULM platform should be leveraged to explore how regional microvascular remodeling contributes to brain function. Specifically, investigating whether changes in hippocampal vascular structure and flow correlate with cognitive decline, and how vascular alterations in white matter may relate to disruptions in large‐scale functional connectivity in murine models of VCID. These efforts will build on the current structural framework and help establish mechanistic links between localized vascular dysfunction and broader neurophysiological outcomes associated with aging and neurodegeneration.

### 3.1 Limitations

While our study provides valuable insights, several limitations should be acknowledged. First, the use of anesthetized animals may influence cerebrovascular dynamics, potentially affecting the generalizability of the findings to awake conditions. Second, the study's cross‐sectional design limits the ability to infer causality between observed vascular changes and cognitive decline. Longitudinal studies are needed to establish temporal relationships. Additionally, while fUS offers high spatiotemporal resolution, it may not capture all aspects of neurovascular function, such as metabolic changes associated with neuronal activity. ​Additionally, while our study provides valuable insights, several limitations should be acknowledged. First, access to raw radiofrequency (RF) data was not available due to technical constraints of the ICONEUS One platform. As such, ULM processing was conducted using image‐based outputs generated by the system's internal beamforming and coherent compounding algorithms. Although RF‐level processing can provide increased flexibility and theoretical gains in spatial resolution, the use of compounded image data for ULM has been well validated in the literature,^[^ ^32^, ^80^ ^]^ and our own prior work^[^ ^34^ ^]^ has demonstrated its reliability in quantifying cerebrovascular remodeling in aging. Nevertheless, future studies incorporating RF‐based custom beamforming may further enhance resolution and localization accuracy. It is important to interpret vessel diameter measurements within the practical resolution limits of ULM. Although MB localization can reach a precision of a few micrometers, the system's effective spatial resolution, particularly in the lateral plane, is limited to ≈10 µm due to the ultrasound beam width and detection constraints as previously discussed.^[^ ^81^ ^]^ Therefore, estimated diameters below 5 µm likely fall below the resolving capacity of the system and should be interpreted with caution. While the current ULM analysis is based on 2D projections over a 500 µm‐thick stack, this approach captures a representative portion of the cortical vasculature. However, we acknowledge the importance of full 3D reconstructions for broader generalization across brain regions. Recent developments in volumetric ULM^[^ ^28^ ^]^ offer promising avenues for future expansion of our methodology. Additionally, we note that our analysis did not stratify vessels by type (capillaries vs arterioles/venules), which may further obscure subtle regional differences in microvascular composition and limit direct anatomical interpretation of size distributions across brain regions. Finally, the exclusive use of male mice limits the generalizability of our findings, as sex differences in cerebrovascular physiology and aging are well documented.^[^ ^82^ ^]^ Future studies should incorporate both sexes to better understand potential sex‐specific differences.

## 4 Experimental Section

### Experimental Design

This study aimed to investigate age‐related changes in cerebrovascular architecture and function in C57Bl/6 mice, focusing on vascular density ratio, CBF, and NVC. Two groups of male mice were used: young (4–6 months) and aged (24 months) C57Bl/6 mice were subjected to fUS imaging of the brain to uncover the cerebrovascular differences occurring with aging. To facilitate the fUS imaging, a TPX window was surgically implanted in place of the skull, ensuring a stable environment for imaging. CBF, NVC, and vascular density ratio were then measured in the two experimental groups. Mice were injected with a microbubble ultrasound contrast agent, consisting of a lipid shell encasing a perfluoropropane gas core, which enhanced the echogenicity of the blood and improved visualization of CBF. All experimental procedures were conducted in accordance with the principles outlined in the “Guide for the Care and Use of Laboratory Animals” published by the National Institutes of Health. For the experiments C5,7BL/6 male mice were purchased from Charles River Laboratories (Wilmington, MA). Animals had ad libitum food (standard rodent chow) and water access in 12–12 h of light‐dark cycle. The animals were housed under specific pathogen‐free conditions until the cranial window surgery in the Rodent Barrier Facility at University of Oklahoma Health Sciences Center (OUHSC), after which they were transferred to the conventional animal facility of the OUHSC and single housed after surgery. The University of Oklahoma Health Sciences Center (OUHSC) Institutional Animal Care and Use Committee (IACUC) committee approved experimental protocols, including consideration of animal welfare and administered drug side effects. All experimentation complied with the ARRIVE guidelines (Animal Research: Reporting in Vivo Experiments) for how to report animal experiments.

### Surgical Window Implantation

The procedure for skull removal was performed under aseptic conditions to minimize the risk of post‐operative infections as previously reported in ref. [34]. Briefly, mice were anesthetized using isoflurane 2% gas via inhalation, and their depth of anesthesia was monitored throughout the procedure. The head of the mouse was fixed in the stereotaxic frame (51 625 W, Stoelting Co, Wood Dale, IL, USA), and the animal's temperature was maintained stable through a heating pad (RT‐0501, Kent Scientific Corporation, Torrington, CT, USA). The head was then shaved, and the scalp was carefully incised along the medial axis, extending from just above the eyes to the base of the skull, and then gently retracted to reveal the underlying skull. A high‐speed microdrill (MicroMotor Technician‐handle – 35 K RPM Polisher Micro Motor Polishing Hand Tool, 110 V, Amazon) was then utilized to thin the skull over the desired region perimeter (6 mm anterior‐posterior and 8 mm medial‐lateral cranial window), taking utmost care to prevent damage to the underlying dura mater. Subsequently, a precise section of the skull was delicately lifted and removed using fine forceps, exposing the brain's surface. Extreme care was taken to delicately remove the skull plate while preserving the integrity of the larger vessels and superior sagittal sinus. Throughout this procedure, ice‐cold sterile saline was intermittently applied to reduce bleeding and swelling, prevent thermal damage, and maintain tissue integrity. An ethanol‐sterilized 4 mm long, 8 mm wide TPX plastic window, commercially known as TPX (film, thickness 0.125 mm, Mitsui Chemicals), was chosen. The window was meticulously sized to fit the craniectomy site and curved using a heated element to mimic the skull curvature. The window was sterilized in 70% ethanol for 10 min before being placed in contact with the brain. Using biocompatible adhesive (Super Glue by Starbond GAP FILLER, Amazon) and sutures (Chromic 5‐0 C6 18 5/0 Chromic Gut Suture, Reliable Dental Supply, Fort Worth, TX, USA) the TPX window was secured in place. The perimeter was sealed with the same adhesive to prevent potential contaminants and ensure the window's stability for subsequent imaging sessions. The animals were then allowed to recover while being monitored. The postoperative care routine for each animal included the administration of Buprenorphine extended release (1 mgkg^−1^, ZooPharm, WY, USA) immediately after the surgery and Baytril 2.27% (10 mgkg^−1^, Elanco Animal Health, IN, USA) once a day for 4 days, including the surgery day.

### fUS Imaging and ULM Imaging

Two weeks after the surgical operation, the animal was lightly anesthetized (1–1.5% isoflurane) and placed on a thermoregulated stereotactic frame (51 625 W, Stoelting Co, Wood Dale, IL, USA). The head of the mouse was shaved using a shaving cream (Nair hair removal body cream, Aloe) and cleaned with ethanol. The ultrasonic probe (IcoPrime‐4D MultiArray 15 MHz, ICONEUS, France) of the Iconeus. One fUS device (ICONEUS, France) was then positioned exactly above the cranial window and immersed in ultrasound gel (Gel de contact, 10 904, Drexco Medical, Crosne, France). fUS data was captured using the scanner's live acquisition software (IcoScan, Iconeus, Paris, France). All fUS data were acquired using a set of ten tilted plane‐wave transmissions (Transmit frequency: 15 MHz, Pulse widths: two cycles per pulse, −12° to +12° tilting angles) compounded with a 5000 Hz pulse repetition frequency (PRF) leading to a 500 Hz ultrasound imaging frame rate. Raw data for each of the four linear arrays were beamformed independently in the receive mode and independently summed coherently in the transmit mode. Each subarray (4 × 64 elements) was tightly spaced with a 2.1 mm distance between subarrays to optimize the field of view while minimizing cross‐talk. The four images were simultaneously obtained from the corresponding four linear arrays at 500 Hz. Each imaging session lasted 1.5–2 h. Subsequently, the animal was closely observed until it fully emerged from anesthesia, ensuring the absence of any abnormal behavior or signs of discomfort. Ultrasound Localization data were captured using the scanner's live acquisition software (IcoScan, Iconeus, Paris, France). Transmit voltage was limited to 8 V (mechanical index of MI = 0.1) to prevent MB destruction during ULM. All ULM data was acquired using a set of five tilted plane‐wave transmissions (Transmit frequency: 15 MHz, Pulse widths: two cycles per pulse, −12° to +12° tilting angles) compounded with a 5000 Hz PRF, leading to a 1000 Hz ultrasound imaging frame rate for a better estimation of higher speeds in larger arteries. ULM Raw data for each of the four linear arrays were beamformed independently in the receive mode and independently summed coherently in the transmit mode.

### ULM Maps Generation

ULM, also referred to as Super‐resolution Ultrasound Localization Microscopy, hinges on the pinpointing and monitoring of individual MBs that were intravenously injected in the imaged animal. In this study, a volume of 100 µl MB solution consisting of 50 µl of sterile saline and 50 µl of sterile MBs suspension (DEFINITY, Lantheus, Billerica, MA, USA) was used to obtain high‐definition images of the overlayed trajectories of each MB in a 500 µm thick coronal brain volume. Before performing the retro‐orbital injection, DEFINITY MBs need to be activated by vigorous agitation for 45 s until the clear solution becomes milky. This MB technique, coupled with the clear TPX window and two weeks of healing time, allowed for reliable capture of high‐resolution (2 µm/pixel) images from the surface to the base of the brain. Acquisitions were performed on an Iconeus One ultrasound imaging system (Iconeus One – 256 channels, ICONEUS, France), equipped with a 15‐MHz MultiArray transducer (IcoPrime‐4D MultiArray, ICONEUS, France). Successive raw ultrasound images were collected for 10 min after the injection of a MBs bolus. ICONEUS software generated a maximum projection of all the MBs trajectories within a single coronal section, exportable in three different images representative of MBs speed, intensity, and Z plane direction. The high‐quality image‐based data exported from the ICONEUS One system were subjected to proprietary internal signal processing. Specifically, the system executes in situ beamforming and coherent plane‐wave compounding before exporting image frames for downstream analysis. In this implementation, multiple ultrasound plane waves were emitted at different angles via electronic beam steering, and the resulting echoes were coherently summed to enhance spatial resolution and signal‐to‐noise ratio.^[^ ^32^, ^80^ ^]^ Although raw RF data were not accessible from the system used in this study, the compounded image frames generated by the ICONEUS software were suitable for MB tracking and super‐resolution vascular mapping, as demonstrated in prior studies using the same imaging pipeline.^[^ ^34^ ^]^

### Image Analysis

All image processing and analysis were done using FIJI v. 1.52p software (Wayne Rasband, National Institutes of Health, USA) open‐source software package, and with MATLAB R2023a version. To obtain the vascular density ratio, grayscale images exported from the ICONEUS software were then converted into binary images with a custom MATLAB script, which converted every colored pixel into a white signal (255). The script also allowed for the selection of ROIs, such as cortex, hippocampus, and white matter, by determining the percentage of positive pixels in that region, representative of the vascular coverage of the region. For the assessment of vascular diameter, we used a FIJI function called “Local thickness”. The exported high‐resolution ULM recordings were processed first. Images were collected from every measurement into a stack and the Weka Trainable Segmentator, a supervised machine learning algorithm in FIJI, was used for batch segmentation to generate binary vascular masks. To estimate vessel diameters, the Local Thickness function implemented in FIJI, based on a previously published method was used.^[^ ^83^ ^]^ This model‐independent algorithm calculates, for each pixel in a segmented binary image, the diameter of the largest circle (in 2D) or sphere (in 3D) that fits entirely within the structure and contains that pixel. The result was a grayscale map where each pixel intensity reflects the local cross‐sectional thickness of the vascular mask. For visualization purposes, a color lookup table was applied to the grayscale maps, but all quantitative measurements were performed on the underlying raw intensity values. Thickness values were extracted pixelwise and used to generate histograms of vessel diameter distribution. Although our exported ULM images had a pixel resolution of ≈2 µm, the Local Thickness plugin performs sub‐pixel interpolation during analysis. However, the effective lateral resolution of ULM was constrained by system‐specific factors such as the point spread function, bubble density, and motion blur. As reported in the literature,^[^ ^81^ ^]^ the effective resolution for structural vessel measurements in ULM was closer to 10 µm. In particular, fUS and ULM imaging sequences implemented on the Multi‐Array probe used in this study had been extensively characterized in a recent work.^[^ ^84^ ^]^ The ULM spatial resolution obtained using the same device, same data processing, and same ultrasonic probe had been estimated using the Fourier Ring Correlation method and determined a resolution limit of 13.33 µm for transcranial ULM imaging in mice. Thus, this resolution limit corresponds to a worst‐case scenario here, as The experiments were performed through TPX chronic windows and did not suffer skull bone aberrations, contrary to this recent study. Accordingly, a conservative minimum reliable vessel radius of ≈5 µm was set. The Allen Brain atlas was superimposed to select the ROIs of interest wisely. The data from every single pixel was exported on an excel sheet to define the diameter distribution within each region. The speed at which the MBs travelled was assessed by exporting high‐resolution images from IcoStudio and translating to grayscale values by a custom MATLAB code. The same ROIs were selected in these images by saving the ones used for the other measurements. The data from every single pixel was exported into Excel to define the speed distribution within each region.

### CBF

To generate spatially resolved, physiologically relevant maps of resting CBF, a custom computational approach was developed that integrates structural and dynamic vascular information derived from ULM and fUS imaging. Specifically, a modified Poiseuille equation was applied on a pixel‐by‐pixel basis, allowing for volumetric flow (Q) estimation across the imaged brain regions. Vessel diameter was determined using the “Local Thickness” algorithm on high‐resolution binary vascular masks generated from ULM images, while blood flow velocity was inferred from grayscale speed maps derived from the movement of intravenously injected MBs tracked by fUS. This integrated analysis yielded quantitative, spatially resolved CBF maps across anatomically defined regions, including the cortex, hippocampus, and subcortical white matter. Applying this method in young and aged mice, significant age‐related reductions in resting CBF were identified within the hippocampus and white matter, consistent with regional vulnerability to microvascular aging and cognitive decline. To achieve this, the original Poiseuille equation was used as the starting point.

Poiseuille equation:

(1) $$Q\left(volumetric\;flow\;rate\right)=\frac{\Delta P\pi r^{4}}{8\mu L}$$

where:

Q = Volumetric flow rate

ΔP = Pressure difference between the two ends of the vessel

 r = radius of the vessel

µ = Dynamic viscosity of the fluid

L = Length of the vessel

The equation can be simplified if blood flow velocity *v* and vessel radius *r* can be estimated:

(2) $$estimated\;Q=v*A=v*\pi r^{2}*K$$

where:

Estimated Q = Calculated volumetric flow rate

 v = Estimated blood flow velocity (MBs velocity in mm/s)

 r = Estimated radius of the vessel (microns)

A = Calculated cross‐sectional area of the vessel

K = 6 × 10^−8^ (allows for conversion of calculated Q units into mL/min)

Subsequently, the estimated Q values were evaluated in specific ROIs (i.e., cortex, hippocampus, and white matter) as the average Q value in the selected area. For the Q distribution, single pixel values were mathematically calculated, following the Poiseuille formula, using the excel sheets obtained from local Thickness and speed data.

### AVR

For a separate assessment of the arterioles and venules in the mouse brain cortex, ULM axial directionality map exports were used. AVR analysis was restricted to the cortical region due to technical limitations in flow direction detection. The current fUS system could only determine blood flow direction along a single axis. In the cortex, this axial sensitivity aligns with the predominant orientation of penetrating arterioles and venules, allowing reliable discrimination between them. Directionality maps had been adjusted to show “y” axis flow orientations: flow to “0” was red, flow from “0” to infinity was blue. In the following, RGB directionality maps went through a color channel split, and only red (arteriolar) and blue (venular) channels were kept for further analysis. The total vascular density ratio between arterioles and venules was measured by a MATLAB script by comparing the number of pixels belonging to arterioles and venules in the cortex as a % of the total vasculature.

### NVC

NVC was evaluated by measuring CBV responses using fUS during repeated whisker stimulation. Unlike the ULM‐based assessment of resting CBF (Figures 4, 5), the fUS approach is optimized for detecting relative changes in blood volume with high temporal resolution. This method enables real‐time tracking of stimulus‐evoked hemodynamic responses in the somatosensory cortex, providing insight into functional vascular reactivity. Anesthetized animals placed on the stereotaxic frame with heat support under the fUS probe were subjected to neurovascular coupling measurements. Isoflurane was lowered to 0.8‐1% maintenance dose to achieve the best results, and anesthesia depth was monitored throughout imaging. Baseline CBF measurements were recorded, followed by sequential whisker stimulation on alternating sides. Whiskers were stimulated for 30‐s intervals with 30‐s rest periods for seven times each side. Changes in CBF were recorded during these stimulations at the level of the contralateral barrel cortex. The acquisition was performed with a minimal allowable pause of 200 ms between scans, providing the necessary high spatial and temporal resolution to effectively capture NVC dynamics. For the performed NVC measurements, fUS imaging was performed using the Iconeus One system equipped with a 15 MHz 4D probe. In this configuration, fUS provides an in‐plane spatial resolution of ≈110–98 µm, an out‐of‐plane (slice thickness) resolution of ≈500 µm, and a maximal temporal resolution of 200 ms. These spatial resolution parameters of fUS imaging were characterized in a very recent study for the same device, same data processing, and same ultrasonic probe.^[^ ^84^ ^]^ This resolution is sufficient to detect regional CBV hemodynamic responses to neuronal activation but did not permit visualization of individual capillaries or small vessels as achieved with ULM.

### Statistical Analysis

All the statistical analysis was performed with OriginLab and GraphPad software. The study was conducted on six young mice and seven aged mice. For the cortex, hippocampus, and white matter, each measurement was made on both sides of the brain. A statistical analysis using a Student's t‐test for unpaired measurements was conducted to compare the means of two independent groups: aged and young individuals. This analysis assumes equal standard deviations (SD) between the groups, which allows for the use of a pooled variance estimate. However, for parameters where unequal variances and potential skewed distributions were observed, specifically, the speed and local thickness measurements, Welch's t‐test was used instead, as it did not assume equal variances and provides a more robust comparison under these conditions. Different P‐values were specified in the figure legend.

## Conflict of Interest

The authors declare no conflict of interest.

## Author Contributions

The study was conceptualized by S.T., S.N., A.C., Z.U., A.Y., and M.T. Methodology was designed by S.T., S.N., and A.N. Investigation and visualization were carried out by S.T., S.N., and R.J. Supervision was provided by S.T., A.C., Z.U., A.Y., and M.T. The original draft was written by S.T., S.N., A.C., Z.U., A.Y., and M.T., while editing was performed by S.N., A.N.T., M.M., E.T.R., S.T., J.I., Z.R., R.R., R.G., R.J., A.C., Z.U., A.Y., and M.T.

## Data Availability

The data that support the findings of this study are available from the corresponding author upon reasonable request.
